# Indirect and direct effects of factors associated with diabetes amongst the rural black population in the Dikgale Health and Demographic Surveillance System, South Africa

**DOI:** 10.4102/phcfm.v13i1.2819

**Published:** 2021-07-15

**Authors:** Peter M. Mphekgwana, Linneth N. Mabila, Eric Maimela

**Affiliations:** 1 Department of Research Administration and Development, University of Limpopo, Polokwane, South Africa; 2 Department of Pharmacy, Faculty of Health Sciences, University of Limpopo, Polokwane, South Africa; 3 Department of Medical Sciences, Faculty of Health Sciences, University of Limpopo, Polokwane, South Africa; 4 Public Health and Health Promotion, Faculty of Health Sciences, University of Limpopo, Polokwane, South Africa

**Keywords:** diabetes, hypertension, cardiovascular diseases, indirect effect, direct effects, rural

## Abstract

**Background:**

Diabetes is an enormous, growing clinical and public health problem, which together with hypertension contributes significantly to the high risk of cardiovascular diseases (CVDs) globally.

**Aim:**

To examine the indirect and direct effects of risk factors simultaneously as a network of multiple pathways leading to diabetes in the rurally based adult population (aged 15+) using a household survey.

**Methods:**

This investigation was based on a predictive model using a cross-sectional community-based study to identify the direct and indirect effects of diabetes risk factors in the Dikgale Health and Demographic Surveillance System (HDSS) consisting of 15 villages, with 7200 households and a total population of approximately 36 000. Fasting blood glucose and total cholesterol were measured using ILAB 300 with the following cut-off values: high fasting blood glucose 7 mmol/L and triglycerides 1.70 mmol/L.

**Results:**

A total of 1407 individuals were interviewed, of whom 1281 had their blood pressure (BP) measured. The conceptual model was validated by the goodness-of-fit indexes (comparative fit index [CFI] = 1.00, Tucker Lewis index [TLI] = 1.041, root mean square error of approximation [RMSEA] = 0.001). Hypertension had the strongest direct effect of 0.0918 on diabetes, followed by age (0.0039) and high waist circumference (−0.0023). Hypertension also mediates the effects that high waist circumference (0.0005) and triglycerides (0.0060) have on diabetes status.

**Conclusion:**

The results in this study confirm the conceptual model considered in the risk factors for diabetes and suggest that hypertension, age and high waist circumference are the key variables directly affecting the diabetes status in the South African rural black population. The direct effect of triglycerides on diabetes suggests mediation by some measured factor(s).

## Introduction

Diabetes is an enormous, growing clinical and public health problem^1^ that together with hypertension contributes significantly to the high risk of cardiovascular diseases (CVDs) globally. In 2012, non-communicable diseases (NCDs) such as diabetes and hypertension were responsible for more than 16 million premature deaths (under the age of 70) worldwide, with diabetes being the fourth leading cause of death in most developed nations.^2,3,4^ In 2019, it is estimated that 9.3% (463 million people) of people aged 20–79 years had diabetes globally and it is projected that the number will reach 578 million by 2030 and 700 million by 2045.^5^ Diabetes is estimated to cause 1.6 million premature deaths globally^5^ and it is the top 10 leading causes of disability-adjusted life-years in age groups 50–74 years and 75 years and older.^6^ The prevalence of diabetes continues to increase more rapidly in low- and middle-income countries than in higher-income countries.^7,8^ People with diabetes are at higher risk of experiencing a variety of complications such as heart disease, stroke, kidney failure, leg amputation, vision loss and nerve injury. Pregnant women with diabetes are at higher risk of foetal death.^7^

The African region has the highest proportion of undiagnosed diabetes; over one-half (232 million) of people with diabetes are unaware if they have the diseaseat all.^9^ In 2015, almost 2.3 million people had diabetes in South Africa, with more than a quarter (38%) of people with diabetes being unaware if they have it all.^9,10^ As per the report by Statistics South Africa (StatsSA) mid-year estimates that the group most affected by the disease is people between the age of 21 and 79 years.^11^ In 2018, the total direct cost attributable to patients who were diagnosed, treated and controlled was over 2.7 billion South African Rand (ZAR), which is equivalent to 198 million US dollars (USD), with half of the cost attributable to treating and managing type 2 diabetes mellitus.^12^

Literature suggests that type 2 diabetes is associated with an interplay of multiple factors such as socio-economical, health behaviours and anthropometric.^13,14^ Socio-economic factors such as income, education, occupation and marital status were found to be associated with diabetes prevalence.^10,13,15^ Indicating that people who are less likely to develop diabetes and to experience its complications are those who earn higher income with the highest level of education attained.

Health behaviours such as smoking, alcohol use, physical inactivity, low fruit and vegetable intake were found to be risk factors associated with diabetes.^10,16^ A large household NCD risk factor survey conducted in the North Indian state of Punjab found that hypertension is significantly associated with diabetes.^17^ Previous studies suggest that diabetes is associated with multiple anthropometric factors such as increasing obesity indexes, body mass index (BMI), waist circumference and waist-height ratio (WHR).^12^ These are much more strongly related to insulin resistance and as such, the higher they are the more prone is an individual to insulin resistance.^18,19^

In an earlier study conducted in Dikgale Health and Demographic Surveillance Site (DHDSS) by Maimela et al., it was found that age and low fruit and vegetable intake are the key variables affecting high fasting blood glucose. The study did not include biomedical risk factors and income as the determinants of fasting blood glucose. Their model, however, relies heavily on the fitting of a logistic model to high versus low fasting blood glucose response variables. This model allows us to treat each covariate in the model that has an independent direct effect on the response variable. Therefore, this study aimed to examine the indirect and direct effects of risk factors simultaneously as a network of multiple pathways leading to diabetes in the rurally based adult population (aged 15+) using a household survey.

## Methods

### Study design and population

The current investigation was based on a predictive model using a cross-sectional community-based study to identify direct and indirect effects of diabetes risk factors in the HDSS, which consists of 15 villages situated close to one another, made up of 7200 households and a total population of approximately 36 000. The population has high rates of illiteracy and unemployment.^13,20^ The study site is located about 30 km north of Polokwane, the administrative capital of the Limpopo province. Ethical approval for the study was obtained from the Medunsa Research and Ethics Committee (MREC) at the University of Limpopo and the Department of Health of the Limpopo province. The Dikgale Tribal Authority permitted the study to be conducted.

### Sampling strategy

Assuming confidence of 95%, a margin of error of 5% and a conservative prevalence estimate of 50%, the initial sample size was set at 380 per age group. No adjustment for design effect was required as, based on the HDSS database, an individual sample frame was used. To ensure a balanced sample of age groups (15–24, 25–34, 35–44, 45–54, 55–64 and ≥ 65 years), we used stratified random sampling. For each age group, the sample size was adapted using a finite population correction (FPC).^17^ A total of 2981 participants were then selected from the HDSS database to take part in the STEPwise approach to chronic disease risk factor surveillance. A total of 1407 people (878 women and 525 men) completed the WHO STEPwise questionnaire, while 331 people were excluded from the analysis as there were missing values on fasting blood glucose. The main reasons for not participating included: participants not being at the home on the day of data collection as the majority works in the city and on surrounding farms and return home on weekends or late in evenings, refusal, death and migration out of the study area. Only 1076 participants were available to donate a fasting blood sample as the others left for work early in the morning. Participants who were HIV-positive were excluded from the biochemical data analysis as HIV patients who are treated with antiretroviral medications (ARVs) develop undesirable changes in lipid and glucose metabolism that mimic the metabolic syndrome.

### Data collection instrument

The field workers were trained by a medical scientist using the World Health Organization’s STEPwise instrument for NCD risk factor surveillance.^21^ After the training, a pilot study was carried out to test the knowledge of the field workers and the feasibility of the data collection tool. Blood pressure was measured three times using the OMRONM6 and M5-I digital automatic BP monitors and the average of the last two readings was used. Diagnosis of hypertension was indicated when systolic BP was ≥ 140 mmHg, when the average diastolic BP was ≥ 90 mmHg or when participants were on anti-hypertensive treatment.^13,22^ Fasting blood glucose and total cholesterol were measured using ILAB 300 with cut-off values of high fasting blood glucose of 7 mmol/L and triglycerides level of 1.70 mmol/L. The prevalence of diabetes mellitus is defined as fasting blood sugar equal to or more than 126 mg/dL. Waist circumference was measured once using a constant tension tape and recorded to the nearest 0.1 cm (high waist circumference > 102 cm for men and > 88 cm for women).^13^ For smoking, the participants were classified as a smoker or non-smoker. For alcohol use, the participants were classified as alcohol drinkers or non-alcohol drinkers.

### Statistical analysis

All statistical analyses were performed using STATA statistical software (STATA Corporation, College Station, Texas). Cross-tabulation analysis, the Chi-square and Fisher’s exact tests were used to test for the differences in socio-behavioural and biochemical variables between participants with diabetes and normal glucose. The study fitted structural equation models with path analysis to the HDSS dataset with 13 observed variables, namely gender, age, marital status, education status, household income, BMI, fasting blood glucose, hypertension, triglycerides and cholesterol, waist circumference, smoking status and alcohol consumption. Variables could have direct, indirect or both effects on the model. One of the advantages of the structural equation model is that it is possible for a variable to not have a direct effect on other variables but to have an indirect effect through other variables. The direct and indirect effects were selected based on the factors previously reported to be associated with diabetes and on expert opinion. Diabetes was defined as the response variable and socio-behavioural and biochemical variables were assumed as exogenous variables. Age was also assumed as an exogenous variable. Hypertension and BMI were added to the model as mediator variables. Fit indices such as Chi-square, comparative fit index (CFI), goodness-of-fit index (GFI), Akaike’s information criterion (AIC), Tucker Lewis index (TLI) and root mean square error of approximation (RMSEA) were measurement of a model good fit. The structural model is a good fit to data when RMSEA < 0.06, CFI > 0.95 and TLI > 0.95.^23^ The model with the lowest AIC value was considered to be the best model.^24^

### Ethical considerations

Ethical approval for the study was obtained from the Medunsa Research and Ethics Committee (MREC) at the University of Limpopo and the Department of Health of the Limpopo province – REC-0310111-031.

## Results

Table 1 presents the results on the characteristics of the population under study.

**TABLE 1:** Characteristics of the study population in Health and Demographic Surveillance System, South Africa.

Variables	Total (1076)		Diabetes (300)		Normal Glucose (776)		Chi-sqaure test /Fisher’s exact test *p*-value
	*n*	%	*n*	%	*n*	%	
**Age (years)**							< 0.001
15–24	295	27	41	14	254	86	-
25–34	151	14	29	19	122	81	-
35–44	102	9	32	31	70	69	-
45–54	144	13	47	33	97	67	-
55–64	157	15	57	36	100	64	-
65+	227	21	94	41	133	59	-
**Gender**							0.012
Male	412	38	97	24	315	76	-
Female	664	62	203	31	461	69	-
**Marital status**							< 0.001
Married or living with partner	395	37	149	38	246	62	-
Never married	586	54	114	20	472	80	-
Divorced or widowed	95	9	37	39	58	61	-
**Education**							0.001
High school or less than	634	59	202	67	432	56	-
More than high school	442	41	98	33	344	44	-
**Household income**							0.001
Less than 2500	706	82	221	31	485	69	-
2500–4500	137	16	23	17	114	83	-
4500–6500	12	2	5	42	7	58	-
> 6500	3	0	0	0	3	100	-
**BMI (kg/m^2^)**							0.003
< 18.5	62	6	17	27	45	73	-
18.5–24.9	460	43	103	22	357	78	-
25.0–29.9	302	28	104		198	66	-
> 30	252	23	76	30	176	70	-
Hypertension (BP ≥ 140/90 mmHg)	440	41	146	49	294	38	0.001
High Triglycerides (≥ 1.7 mmol/L)	124	23	46	29	78	21	0.049
High HDL cholesterol (≥ 5.0 mmol/L)	172	32	62	39	110	30	0.038
High waist circumference (≥ 102 cm men and ≥ 88 cm women)	329	31	98	33	231	30	0.355
Current smoker	929	86	265	88	664	86	0.236
Alcohol use	892	83	256	85	636	82	0.187

BP, blood pressure; BMI, body mass index; HDL, high-density lipoprotein.

We then determined the coefficient of the independent variables to establish the direct, indirect and total effects on diabetes for adults aged 15–98 years in the study site. The result for this process are presented in Table 2.

**TABLE 2:** Direct, indirect and total effects on diabetes for adults aged 15–98 years in Health and Demographic Surveillance System, South Africa.

Variable	Direct effect	Coefficient (*p*-value)	Indirect effect	Coefficient (*p*-value)	Total effect	Coefficient (*p*-value)
Age	0.0039	< 0.001	0.0001	0.893	0.0039	< 0.001
Smoke status	-	-	0.0012	0.912	0.0012	0.912
Alcohol use	-	-	0.0009	0.912	0.0008	0.912
Marital status	-	-	−0.0052	0.170	−0.0052	0.170
Education	-	-	−0.0096	0.140	−0.0096	0.140
BMI	0.0002	0.922	0.0001	0.890	0.0002	0.912
Hypertension	0.0918	0.024	-	-	0.0918	0.024
TG	0.0206	0.420	0.0060	0.098	0.0267	0.297
WC	−0.0023	0.035	0.0005	0.044	−0.0019	0.088

BMI, body mass TG, Triglyceride; WC, waist circumference.

**FIGURE 1:**
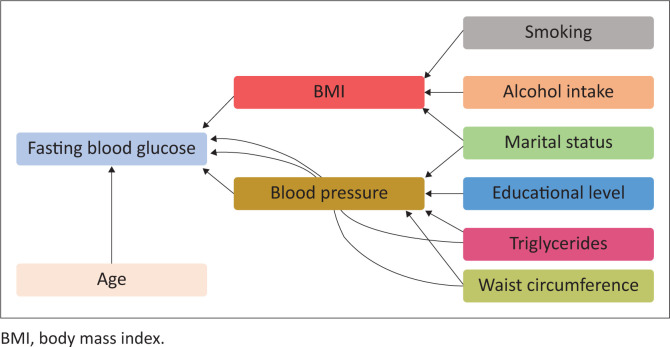
Indirect and direct effects of risk factors for diabetes in Health and Demographic Surveillance System, South Africa amongst adults aged 15–98 years.

To fit a structural equation model, all the categorical independent variables have been modified to a conventional measurement model for continuous indicators, which would yield a single coefficient. However, for continuous variables, the structural model remained essentially the same. The results of this modification are illustrated in Table 3 and Figure 1.

**TABLE 3:** Coefficients obtained by Structural Equation Modeling for adults aged 15–98 years in Health and Demographic Surveillance System, South Africa.

Variables	Coefficient	Standard error	*P*-value	95 % CI	
				Lower	Upper
**BMI**					
Age	0.0545	0.0578	0.346	−0.0587	0.1678
Smoking	0.1912	0.0443	**< 0.05**	0.1043	0.2782
Alcohol use	0.1529	0.0446	**0.001**	0.0654	0.2404
Marital Status	−0.0887	0.0533	**0.096**	−0.1932	0.0157
Education	−0.0530	0.0475	0.265	−0.1463	0.0402
**Hypertension**					
Age	0.0060	0.0597	0.920	−0.1110	0.1231
Marital status	−0.1069	0.0537	**0.047**	−0.2123	−0.0015
Education level	−0.0989	0.0479	**0.039**	−0.1928	−0.0049
BMI	0.0058	0.0422	0.890	−0.0768	0.0885
WC	0.1834	0.0405	**0.001**	0.1039	0.2630
TG	0.1065	0.0435	**0.014**	0.0211	0.1920
**Diabetes**					
Age	0.1728	0.0438	**< 0.001**	0.0868	0.2588
BMI	0.0041	0.0426	0.922	−0.0793	0.0876
Hypertension	0.0988	0.0434	**0.023**	0.0135	0.1840
TG	0.0360	0.0446	0.419	−0.0514	0.1235
WC	−0.0903	0.0426	**0.034**	−0.1740	−0.0066

Note: Bold values represent *p*-values < 0.05.

Chi-square: 7.21; CFI: 1.000; TLI: 1.041; *p*-value: 0.6152. Lower bound: 0.001; RMSEA: 0.0001; *p*-value: 0.982. Upperbound: 0.042.

CI, confidence interval; BMI, body mass index; CFI, comparative fit index; TLI, Tucker Lewis index; RMSEA, root mean square error of approximation; TG, Triglyceride; WC, waist circumference.

## Discussion

A total of 300 out of 1076 study participants had diabetes (28%). The prevalence of diabetes varied according to age group, gender, marital status, level of education, household monthly income, hypertension, triglycerides and density lipoprotein. The proportion of diabetes increased with advancing age (*P*-value < 0.001). Females had a significantly higher prevalence for diabetes 31% as compared with 24% for males (*P*-value = 0.012). The prevalence of diabetes was found to be significantly lower amongst single respondents (20%) as compared with the other groups (*P*-value < 0.001). Participants with a BMI score between 25.0 and 29.9 showed a slightly higher incidence of diabetes relative to other groups (*P*-value = 0.003). Other factors such as: monthly household income, hypertension, high triglycerides (≥ 1.7 mmol/L) and high-density lipoprotein (HDL) cholesterol (≥ 5.0 mmol/L) were also found to be significantly associated with diabetes at a 5% significant level as shown in Table 1. As for high waist circumference, smoking and alcohol use, there was no statistically significant association with diabetes.

The results in Tables 2 and 3 present standardised factor loading values for each model, as well as their significance. Non-significant paths were removed and a few additional paths were added to improve model fit. Specifically, education, marital status, smoking status and serum cholesterol were dropped from the diabetic model for a better fit. The following risk factors are deemed to have a direct, statistically significant effect on diabetes status: increasing age, BMI, hypertension and waist circumference (Figure 1 and Table 2). The indirect effects on the diabetic status of age, smoking, alcohol use, marital status and education status that passes through BMI were not statistically significant. On the other hand, waist circumference and TG were deemed to have an indirect statistically significant effect on diabetes status through hypertension, whereas the variables: age, BMI, smoking; alcohol use, marital status and education status were found not to be statistically significant indirectly effecting diabetes.

The goodness-of-fit statistics of the model are shown in Table 3. The *p*-value for the Chi-square test was greater than 0.05 for the model, suggesting that the model is a better fit for the observed data. However, the Chi-square statistic obtained from structural equation modelling is sensitive to sample size and we, therefore, rely on other criteria, such as CFI, TLI and RMSEA, to determine model goodness fit. The RMSEA value was less than 0.05, while the CFI and TLI values were greater than 1.000, indicating a model goodness fit.

The results in Table 3 showed that age and education level were not statistically significant predictors of BMI. As for the statistically significant variables at the 0.05 level, smoking and alcohol use had the lowest *p*-values, suggesting a strong association with BMI. However, marital status was only significant at the 0.10 significance level. The effect of high waist circumference and triglycerides on hypertension was statistically significant with a positive coefficient, whereas marital status and education level were also statistically significant with a negative coefficient. The parameter coefficient suggests that marital status and education level are factors that decrease the risk of hypertension. The effect of age and hypertension on the risk of diabetes was statistically significant with a positive coefficient. This suggests that hypertension is a factor that increases the risk of diabetes. The effect of waist circumference on the risk of diabetes status was statistically significant with a negative coefficient suggesting a decrease in the risk of diabetes with an increase in waist circumference.

To the best of our knowledge, this is the first comprehensive study in the Dikgale HDSS to identify direct and indirect risk factors for diabetes in a black rurally based population. In this population-based study, the overall prevalence of diabetes was 28%. A study by Mohamed reported no significant gender difference in diabetes.^25^ The present findings contradict the previous findings as significant gender difference was found. Our findings also lend support to previous studies, which indicated that there were more diabetic women than men in the rurally based population.^26,27^ The reason for this difference may be the fact that the study by Mohamed was looking at both urban and rural residences.

Supporting the previous literature,^28^ this study has demonstrated a significant association of marital status with diabetes. A similar conclusion was drawn from the study by LaCoe-Maniaci, who found that diabetes prevalence was significantly higher in those who were widowed, compared with those who were married, and in men, as compared with women.^29^ Diabetes mellitus was most prevalent in the oldest age group (age more than 60 years, 22.9%) and low education groups.^30^ Our findings also lend support to previous studies, which have indicated that diabetes was prevalent amongst those with high school or less than high school education levels.

Previous studies reported^26,31^ hypertension as the most prevalent comorbidity amongst rural and semi-rural patients admitted with diabetes.^15^ The present study also suggests a statistically significant association between hypertension and diabetes, with hypertension having a direct effect (rather than an indirect effect) on fasting blood glucose. Body mass index was also found to have a direct effect on diabetes status amongst the black rurally based population aged more than 15 years old. This finding is consistent with prior research.^15,32,33^ The study by Amiri et al. reported a direct effect of triglyceride levels and BMI on diabetes status.^33^ However, this was not the case in this study, which found that the triglyceride level is indirectly associated with diabetes through BP. A probable reason accounting for these differences is that the study by Amiri et al. stratified the analysis by gender.^33^

In agreement with findings from previous studies that found significant associations between diabetes and waist circumference,^20,34^ this study found that there was a significant, direct and indirect relationship between waist circumference and diabetes. The study by Tripathy et al. reported that the indirect effects of waist circumference, alcohol use and age on blood sugar levels were mediated by raised BP.^20^ Our findings also lend support to previous studies that indicated that BP mediates the indirect effects of waist circumference on blood sugar levels. However, in this study, we could not find any indirect effects of alcohol use on diabetes mediated by raised BP. One possible explanation for these disparities may be the ages of subjects involved in two studies, as this research was aimed at old and young people (less than 18 years of age and greater than or equal to 65 years of age) while the sample by Tripathy et al. participants were between 18 and 69 years of age.

Despite the study’s strength, including a large sample, limitations should be noticed. The survey was cross-sectional and was not conducted throughout the year. This means that no causal relationship can be inferred between any of the factors listed and diabetes. Fasting blood glucose identified by diabetes was measured at a particular point in time when, in fact, it would normally fluctuate. The study acknowledges the limitation posed by the use of structural equation modeling (SEM) in the handling of categorical independent variables.

## Conclusion

This study provides a reference to the prevalence of diabetes in a rural population of South Africa. The findings of our study demonstrated that a considerable proportion of the participants in HDSS had diabetes, indicating a village-level burden. A structural equation modelling identified age, hypertension, BMI and waist circumference as the key variables that directly affect diabetes in this rural South African population. On the other hand, the indirect effects of waist circumference and triglyceride levels on diabetes are mediated by BP. Based on these findings, it is suggested that screening and monitoring for BP and blood sugar levels amongst the rural South African population should be encouraged.
